# Hypoxic Tumor Environments Exhibit Disrupted Collagen I Fibers and Low Macromolecular Transport

**DOI:** 10.1371/journal.pone.0081869

**Published:** 2013-12-12

**Authors:** Samata M. Kakkad, Marie-France Penet, Alireza Akhbardeh, Arvind P. Pathak, Meiyappan Solaiyappan, Venu Raman, Dieter Leibfritz, Kristine Glunde, Zaver M. Bhujwalla

**Affiliations:** 1 JHU ICMIC Program, Division of Cancer Imaging Research, The Russell H. Morgan Department of Radiology and Radiological Science, The Johns Hopkins University School of Medicine, Baltimore, Maryland, United States of America; 2 Sidney Kimmel Comprehensive Cancer Center, The Johns Hopkins University School of Medicine, Baltimore, Maryland, United States of America; 3 Universität Bremen, Fachbereich 2, Bremen, Germany; University of Chicago, Department of Medicine, United States of America

## Abstract

Hypoxic tumor microenvironments result in an aggressive phenotype and resistance to therapy that lead to tumor progression, recurrence, and metastasis. While poor vascularization and the resultant inadequate drug delivery are known to contribute to drug resistance, the effect of hypoxia on molecular transport through the interstitium, and the role of the extracellular matrix (ECM) in mediating this transport are unexplored. The dense mesh of fibers present in the ECM can especially influence the movement of macromolecules. Collagen 1 (Col1) fibers form a key component of the ECM in breast cancers. Here we characterized the influence of hypoxia on macromolecular transport in tumors, and the role of Col1 fibers in mediating this transport using an MDA-MB-231 breast cancer xenograft model engineered to express red fluorescent protein under hypoxia. Magnetic resonance imaging of macromolecular transport was combined with second harmonic generation microscopy of Col1 fibers. Hypoxic tumor regions displayed significantly decreased Col1 fiber density and volume, as well as significantly lower macromolecular draining and pooling rates, than normoxic regions. Regions adjacent to severely hypoxic areas revealed higher deposition of Col1 fibers and increased macromolecular transport. These data suggest that Col1 fibers may facilitate macromolecular transport in tumors, and their reduction in hypoxic regions may reduce this transport. Decreased macromolecular transport in hypoxic regions may also contribute to poor drug delivery and tumor recurrence in hypoxic regions. High Col1 fiber density observed around hypoxic regions may facilitate the escape of aggressive cancer cells from hypoxic regions.

## Introduction

Tumors display abnormal physiological environments such as hypoxia, which primarily arise from their abnormal and chaotic vasculature [1]. Hypoxia is associated with increased resistance to radiation and chemotherapy, and with a more aggressive phenotype [1]. The discovery of the hypoxia inducible factor (HIF), and the identification of hypoxia response elements (HREs) as transcriptional controls in multiple genes [2], is continuing to unravel the critical role of hypoxia in influencing cancer progression and metastasis. The molecular mechanisms underlying the cascade of changes induced by hypoxia and the HIF-axis have attracted significant attention [2], but the functional impact of hypoxia on the tumor extracellular matrix (ECM) and on the transport of macromolecules in the tumor interstitium is relatively unexplored. Our purpose here was to investigate the role of hypoxia in altering the ECM, particularly the collagen 1 (Col1) fiber distribution, and its effect on macromolecular transport. To study the relationship between hypoxia, macromolecular transport, and Col1 fiber distribution, we combined dynamic magnetic resonance imaging (MRI) of the macromolecular contrast agent (MMCA) albumin-Gd-diethylenetriaminepentaacetate (albumin-GdDTPA) to detect interstitial macromolecular transport, with second harmonic generation (SHG) microscopy to measure Col1 fibers morphology and distribution. SHG is a nonlinear optical process that requires a molecular environment without a center of symmetry, such as an interfacial region, to produce a signal that can be used to image endogenous structural proteins such as Col1 [3]. These studies were performed with MDA-MB-231 human breast cancer xenografts genetically engineered to express tdTomato red fluorescent protein (RFP) under control of HRE [4].

As an abundant stromal component, Col1 fibers form a major part of the breast tumor ECM [5]–[8]. Malignant breast cancers are characterized by significantly higher Col1 fiber density and altered Col1 fiber architecture [8]. High mammary Col1 density was shown to cause mammary tumor initiation, progression, and metastasis [8]. We have previously shown that hypoxic regions in breast and prostate tumor xenografts contain significantly lower Col1 fiber density and volume compared to normoxic tumor regions [9]. Hypoxia stimulates the gene expression of a cluster of hydroxylases necessary for Col1 fiber formation [10]. Hypoxic environments in tumors may lead to abnormal collagen deposition either by cancer cells or fibroblasts present within the tumor stroma [8], [11]. In normal tissue, Col1 fibers direct interstitial fluid into lymphatic channels [12]. In tumors, these fibers may not be structured for efficient flow of fluid, especially in hypoxic areas [9]. Here, by careful co-registration of *in vivo* MRI to *ex vivo* optical images, we related macromolecular transport to Col1 fiber morphology and hypoxia. Macromolecules that extravasate from the vasculature into the tumor interstitium, are transported through the ECM either by diffusion or convection [13]–[15]. To understand drug delivery through the tumor ECM, it is important to understand the role of both, Col1 fibers and hypoxia, in this transport.

The movement of macromolecules through the ECM can also provide insight into the movement of metastatic cells through the ECM. Our data suggest that collagen fibers mediate macromolecular transport that is reduced in hypoxic low Col1 fiber containing regions. Around these severely hypoxic regions, a dense Col1 fiber mesh is observed that aligns with increased macromolecular transport. These results have significant implications for the delivery of macromolecular therapeutic agents as they uncover a new aspect of hypoxic environments in limiting macromolecular transport. The sparser fibers within severely hypoxic regions, and the previously observed close correlation between dense collagen fibers and metastasis [16], suggest that the stimulus for invasion may occur from hypoxic environments and the denser collagen fibers around these regions may provide avenues of escape and dissemination in the metastatic cascade.

## Materials and Methods

### Ethics Statement

All experimental animal protocols were approved by the Institutional Animal Care and Use Committee of the Johns Hopkins University School of Medicine under the animal protocol number MO11M122 and under the Animal Welfare Assurance Number A3272-01.

### Tumor Model and Inoculation

The triple-negative metastatic human breast cancer cell line MDA-MB-231 [17] was obtained from the American Type Culture Collection (ATCC, Rockville, MD). To generate MDA-MB-231 cells that express red fluorescent protein (RFP) under hypoxic conditions, MDA-MB-231 cells were stably transfected with a construct containing five copies of the HRE of the human VEGF-A gene ligated to the cDNA of the tdTomato RFP, which produced MDA-MB-231-5HRE-TdTomato cells as previously described and validated [4], [18]. Two million MDA-MB-231-5HRE-TdTomato cells were orthotopically inoculated in the right upper thoracic mammary fat pad of anesthetized female severe combined immunodeficient (SCID) mice. MDA-MB-231-5HRE-TdTomato tumor xenografts reached their final experimental size of approximately 400–500 mm^3^ within 8 weeks and the studies were performed with 10 mice.

### MRI Acquisition

**Figure 1:**
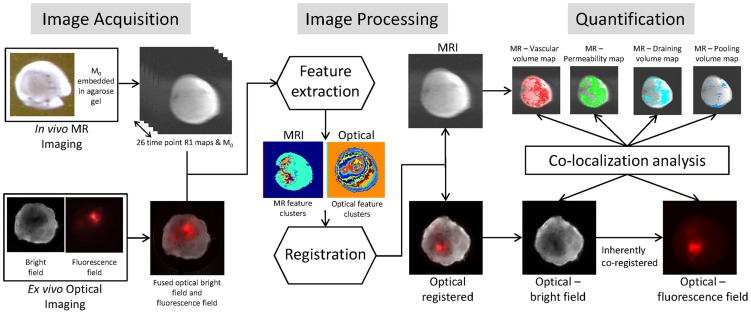
Workflow of the experimental design. The workflow consisted of three distinct parts: image acquisition, image processing and image quantification. For the image acquisition part, we performed *in vivo* MRI to extract functional information of vasculature and macromolecular transport within the tumor ECM. Following MRI, the tumor was excised and *ex vivo* optical imaging performed to extract the distribution of hypoxic regions and Col1 fibers. In image processing, MRI and optical images were co-registered following which vascular and transport parameters in hypoxic and normoxic regions were computed. Col1 fiber distributions in these regions were quantified to give fiber volume and inter-fiber distances. For image quantification, the quantitative data were analyzed and comparisons performed between hypoxic and normoxic regions.

Mice were imaged 8 weeks post inoculation, once tumor volumes were approximately 400–500 mm^3^. Mice were anesthetized for MRI, and a home-built catheter was inserted in the tail vein to deliver the MMCA albumin-GdDTPA. MRI was performed on a 4.7T Bruker spectrometer using a home built solenoid coil placed around the tumor. The respiration rate was monitored, and an isoflurane mask was used to maintain stable anesthesia during the 140 min of MRI scan time. The MRI acquisition was performed as previously described [19]. Briefly, multi-slice relaxation rates (T_1_^−1^) were acquired using a saturation recovery technique with fast-T_1_ SNAPSHOT FLASH imaging (flip angle = 10 degrees, echo time = 2 ms). Images of the central 4 slices (slice thickness of 1 mm) of the tumor were acquired (128×128 matrix, 16 mm×16 mm field of view, number of average = 8) for three relaxation delays (100, 500 and 1000 ms). A multi-slice map of completely relaxed magnetization (M_0_ map) was also acquired with a recovery time of seven seconds. The in-plane resolution of the MR images was 125 µm×125 µm. Interstitial transport parameters were measured from quantitative T_1_ maps obtained before and following intravenous administration of the MMCA albumin-GdDTPA (500 mg/kg dose). Images were acquired in two “phases” corresponding to the biphasic kinetics of the MMCA [19]. The “early phase” acquisition images included a pre-contrast image, and a 3 minute post-contrast image that was repeated every 5 minutes over the initial 30 minutes to characterize the tumor vascular volume (VV) and permeability surface area product (PS). Since drainage of macromolecules in and around tumors either by convection or by the lymphatics is a slow event, the second block of MR data was acquired up to 140 minutes post-contrast and was used to characterize the interstitial transport parameters through the ECM. Transport parameters calculated included number of draining and pooling voxels, draining and pooling rates, and exudate volumes, derived as previously described [19], and briefly outlined in the Quantification section. At the end of the MRI acquisition, blood T_1_ was determined from 20 microliters drawn from the tail vein. The experimental workflow is outlined in Figure 1.

### Fluorescence and Multiphoton Microscopy

Following the MRI acquisition, mice were euthanized and the tumors were excised. The orientation of the tumor in the MRI scanner bore was registered using spatial markers, and the tumor sliced into 1 mm thick sections. Optical imaging of the fresh tissue slices corresponding to the MR-imaged slices was performed to detect hypoxic and normoxic regions. First, an overview of the entire tumor slice was acquired at 1× magnification using a Nikon inverted microscope equipped with a Nikon Coolpix digital camera (Nikon Instruments, Inc., Melville, NY, Figure 1, Image acquisition block – *Ex-vivo* imaging). Images of all four slices were acquired in the bright field for tissue architecture and in the red fluorescence field for detection of hypoxic regions. Optical images acquired with the 1× objective were used to map the MRI data to hypoxic and normoxic regions within the corresponding slices, and identify fields of view (FOVs) to image Col1 fibers at higher magnification. We used multiphoton microscopy to detect the SHG signal from the Col1 fibers in these fresh tissue slices. SHG imaging was performed as previously described [9], [16]. Briefly, we used a 25×/0.8 LD LCI PlanApo multi-immersion lens on a Zeiss 710 LSM NLO Meta multiphoton microscopy system (Carl Zeiss MicroImaging, Inc, Thornwood, NY). The system was equipped with a 680–1080 nm tunable Coherent Chameleon Vision II laser (Coherent, Inc, Santa Clara, CA) with automated pre-compensation and fast scanning at 40 nm/s. 3-dimensional (3D) image stacks were acquired from various FOVs in the hypoxic and normoxic regions. Hypoxic FOVs were selected to contain more than 80% of red fluorescing cells and were therefore severely hypoxic. Normoxic FOVs contained less than 1% of red fluorescing cells. Col1 fibers were illuminated with incident laser light of 880 nm and detected at 410–470 nm. The fluorescence from TdTomato red was excited at a wavelength of 543 nm, and detected at 570–620 nm [9].

Following optical imaging, 5 µm thick-sections were obtained from the optical slice and stained with hematoxylin and eosin stain (H&E) to identify necrotic regions.

### Quantitative Image Analysis

Macromolecular transport in tumors was quantified from the dynamic MR images based on the biphasic kinetics of the MMCA as previously described [19]. Vascular parameters that include VV and PS were calculated from the first 30 minutes during the ‘early phase’. ‘Late phase’ images acquired up to 140 minutes post contrast injection gave macromolecular transport parameters that included draining rate, draining volume, pooling rate, and pooling volume. During the ‘late phase’ a draining voxel was defined as a voxel in which the contrast agent accumulated at a rate lower than the PS, and a pooling voxel was one in which the contrast agent accumulated at a rate higher than the PS. After identifying the draining and pooling voxels, the rates and exudate volumes were calculated. All quantification analysis was done in a home-built program written in IDL (ITT Exelis Visual Information Solutions, VA USA) and AFNI [20].

The structural information of the tumor microenvironment was obtained by quantifying the Col1 fiber distribution acquired with SHG microscopy. Col1 fiber distribution analysis was performed as previously described [9]. Briefly, the inter-fiber distance and percent fiber volume present in hypoxic and normoxic FOVs were quantified. Quantification of the Col1 fiber distance distribution and fiber volume was performed using a customized program written in Matlab (MATLAB 7.4.0, The MathWorks, Natick, MA). Regions of interest (ROIs) containing more than 80% of red fluorescing cells, considered severely hypoxic, and regions with less than 1% of red fluorescing cells, considered normoxic, were analyzed [9]. A two-sided paired t-test (α = 0.05) was used to detect significant differences in inter-fiber distances and percent fiber volumes between these severely hypoxic and normoxic ROIs using Microsoft Office Excel 2010 (Microscoft, Redmond, WA). *P-values* of ≤0.05 were considered to be significant.

To combine functional and structural information, MRI and 1× optical images were co-registered (Figure 1, Image processing and quantification block) to calculate transport parameters in hypoxic and normoxic ROIs. To account for tissue deformation and differences in spatial information we used a feature based co-registration approach, and calculated Dice similarity statistics to quantify the quality of the co-registration [21].

To establish the orientation of the tumor during the *in vivo* MRI acquisition, the mouse was anesthetized and the entire tumor was embedded in agarose gel as shown in Supplementary Figure S1. During agarose embedding the gel was prepared in liquid form (at temperature ∼65°C), which solidified at room temperature around the tumor (Figure S1A). Asymmetrical cuts were made in the surrounding agarose gel before acquiring the MR images. Since agarose gel contains water, it results in a uniform signal around the tumor in the M_0_ map (Figure 1, Image Acquisition block). The asymmetrical markings were visible in the MR images and helped establish the orientation of the tumor within the magnet (Figure S1B). Because of tumor warming that occurred while pouring the liquid gel, and the slight contraction of the gel over the ∼2 h period of imaging, this procedure was only used to establish the orientation of the tumor in the magnet to the images. Data from these tumors were not included to avoid any effects that may have occurred due to heating and pressure on the tumor. Once this was done, all mice and tumors were positioned reproducibly. Following MRI, the tumor was excised and sliced with the tumor orientation maintained as it was in the MRI scanner. For each modality we combined the various images acquired, extracted features, and used the mutual information approach for co-registration between modalities (Figure 1, Image Processing block). For the MR images we used the R1 maps acquired over 26 time points to identify cluster features using the fuzzy c-means (FCM) [22], [23] clustering technique (Figure 1, Image Processing block). For the optical images, we fused the bright field and fluorescent images by wavelet based image fusion [24], and extracted features for the optical images using FCM clustering. The co-registration was semi-automated by FCM clustering that identified features in each image. These features were then used to pick out the registration points for affine transformation. Extracted features from the optical images were registered to the extracted features from the MR image using affine transformation [25]. Rigid-body registration was used to avoid any feature distortion. Since the bright field image and fluorescence field image are intrinsically registered to the fused image, the same transformation matrix was applied to register these images to the MR images. The Dice similarity index [26] was calculated, based on segmented features obtained from two modalities, to assess the registration error. The Dice similarity index (D) is a statistical measure used to calculate the similarity between two data sets. It is calculated by:
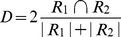



In our study, R_1_ and R_2_ were feature regions from the two modalities (MRI and optical or optical and histology) that were compared [21], [27]. Dice similarity indices ranged from 0 to 1, where 0 indicates no overlap and 1 indicates complete overlap [26]. For multimodality co-registration of *in vivo* MRI and *ex vivo* optical data, a Dice similarity index >0.85 was considered as good overlap [27]. Hypoxic regions were mapped on the MR images using the co-registered optical fluorescent images, to determine the VV and PS values in these regions. To visualize the hypoxic mask, we included all voxels with signal intensity higher than 10% of the maximum fluorescence signal intensity in each optical image. This was done to avoid using a hard threshold, and to exclude any background signal due to autofluorescence. Similarly, the macromolecular transport parameters of draining rate and volume, and pooling rate and volume were calculated for hypoxic and normoxic regions (Figure 1, Image processing and quantification block – Quantification). The co-registration and co-localization analysis was performed in MATLAB. A two tailed t-test was performed using Microsoft Office Excel 2010. *P-values* of ≤0.05 were considered to be significant (N = 10).

The histology sections were co-registered using the same protocol used for the optical and MR image co-registration. The orientation of the histology sections was identical to the optical images. The affine transformation was used to co-register the histology sections to the optical images, and the Dice similarity index was calculated to evaluate the co-registration efficiency.

## Results

**Figure 2:**
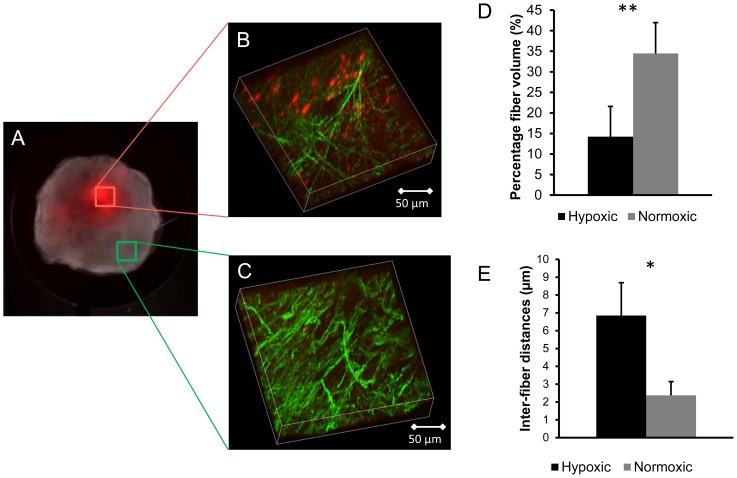
Optical imaging and quantification of fiber volume and fiber distribution. (A) *Ex vivo* 1× bright field and fluorescence image overlaid showing the locations of hypoxic ROIs on the tumor section. 3D visualization of (B) hypoxic and (C) normoxic FOVs; hypoxic regions are displayed in red and Col1 fibers in green. The FOV image size was 334.91×334.91×60 µm^3^ with a voxel size of 0.66×0.66×1 µm^3^. (D) Hypoxic FOVs showed a significantly lower fiber volume as compared to normoxic FOVs (**p-value = 0.000106, N = 10). (E) Hypoxic FOVs have a significantly larger inter-fiber distance as compared to normoxic FOVs (*p-value = 0.000121, N = 10).

Hypoxic regions were identified by tdTomato red fluorescence in the optical images (N = 10, Figure 2A). Using SHG microscopy, we observed significantly fewer and sparsely distributed Col1 fibers in severely hypoxic regions as compared to normoxic regions. A 3D visualization of the Col1 fiber in these hypoxic regions (Figure 2B) and normoxic regions (Figure 2C) revealed that the pockets in the sparsely distributed Col1 fibers were filled with hypoxic cells. Quantitative analysis of the Col1 fibers showed that the percentage of Col1 fiber volume in severely hypoxic regions was significantly lower than that in the normoxic regions (*p-value* = 0.000106, N = 10, Figure 2D). Similarly, the inter fiber distance in these hypoxic regions was significantly greater than that in normoxic regions (*p-value* = 0.000121, N = 10, Figure 2E), indicating more sparsely distributed Col1 fibers in the hypoxic regions as compared to the normoxic regions.

**Figure 3:**
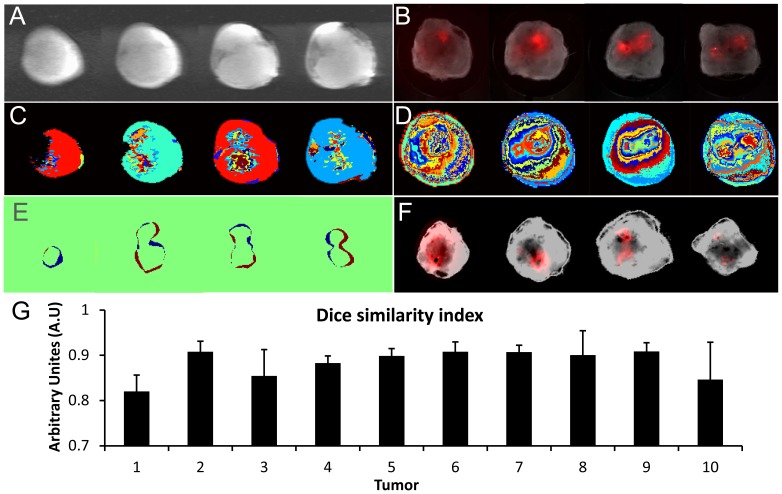
Co-registration results. (A) MR anatomical M_0_ images of the central four slices of the tumor as reference images (FOV = 16×16×4 mm^3^). (B) Display of the optical bright field image overlaid with the fluorescence field image. Features extracted from (C) MR images and (D) optical images, respectively. (E) Display of the extracted features from the MR images overlaid with the optical images after co-registration. (F) Registered optical bright field images overlaid with the fluorescence field images. (G) The co-registration error represented by the Dice similarity index for all tumors (N = 10) in this study.

To understand how these differences in the Col1 fibers functionally affect molecular transport through the tumor ECM, the optical images were co-registered to the corresponding MR sections. Figure 3A shows the M_0_ map of the central of four slices through the tumor. The corresponding fluorescence images overlaid on the bright field optical images before co-registration are displayed in Figure 3B. Figures 3C and 3D show the feature clustering from the MR images and the fused optical images (bright and fluorescence field images), respectively. These extracted features were used to co-register the optical images to the corresponding MR sections. The extracted optical imaging features were co-registered to the extracted MRI features as shown in Figure 3E. The difference in color is the error of registration mismatch between the MR and optical images. Figure 3F shows the co-registered optical (fluorescence image overlaid with the bright field images) and MR images. The registration error between the MR and optical images calculated as the Dice similarity index is shown in Figure 3G for all tumors. The indices were greater than 0.85, indicating successful co-registration between the MRI and optical images.

**Figure 4:**
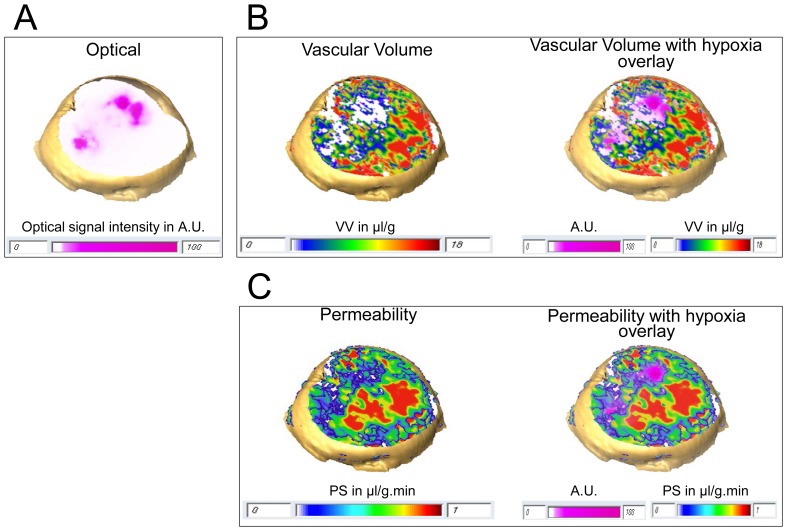
Representative 3D reconstructed view of vascular parameters. 3D reconstruction of (A) optical image with hypoxic regions displayed in pink within the tumor, (B) corresponding display of VV (left), and overlay of hypoxia mask on VV (right), (C) corresponding display of PS (left) and overlay of hypoxia mask on PS (right) in a breast cancer xenograft. To visualize the hypoxic mask, we included all voxels with signal intensity higher than 10% of the maximum fluorescence signal intensity in each optical image. This was done to avoid using a hard threshold and to exclude any background signal due to autofluorescence.

**Figure 5:**
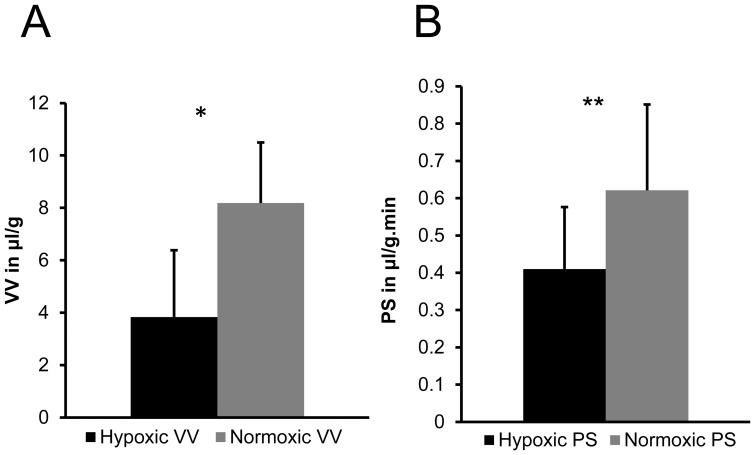
Quantification of vascular parameters. Quantitative comparison of (A) VV and (B) PS for hypoxic and normoxic breast tumor regions. Columns: Mean±standard deviation (SD). We observed significantly lower VV (*p-value = 0.000374, N = 10) and PS (**p-value = 0.000215, N = 10) in hypoxic regions compared to normoxic regions. Columns: Mean±SD.

From the co-registered optical and MR imaging data, we observed that red fluorescing hypoxic regions, displayed in pink to avoid overlap with the MR parametric map color scale, co-localized with regions where the VV was low, as shown by 3D reconstruction of all four slices of the optical and VV data of a representative tumor in Figures 4A and 4B respectively. We also observed lower PS in hypoxic regions as compared to normoxic regions, as seen in Figure 4C. We quantified these parameters and found that VV in hypoxic regions was significantly lower than that in normoxic regions (*p-value* = 0.000374, N = 10, Figure 5A). Similarly, PS values in the hypoxic regions were significantly lower than those in normoxic regions (*p-value* = 0.000215, N = 10, Figure 5B).

**Figure 6:**
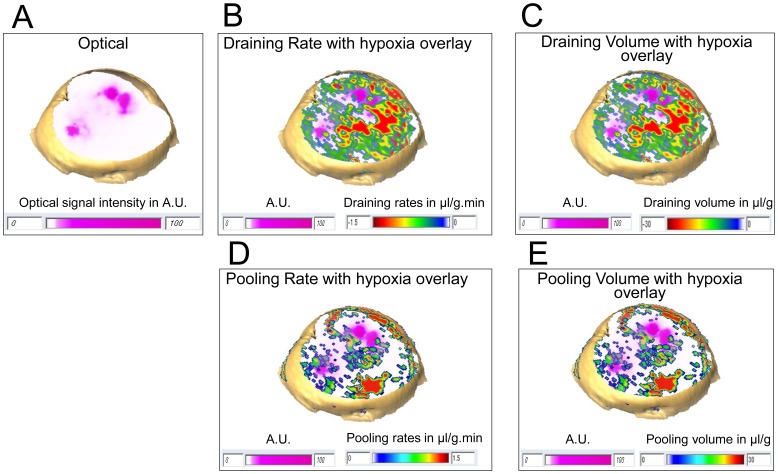
3D reconstructed view of transport parameters from the tumor shown in Figure 4. 3D reconstruction of (A) the optical image as in Figure 4A with hypoxic regions displayed in pink within the tumor, (B) corresponding draining rate, (C) draining volume distribution, (D) pooling rate and (E) pooling volume distribution in a breast cancer xenograft, overlaid with hypoxia mask.

**Figure 7:**
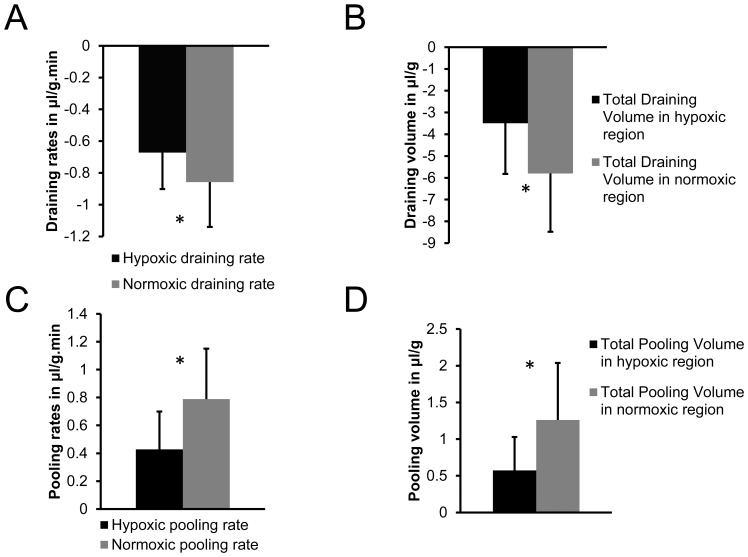
Quantification of transport parameters. Quantitative comparison of (A) draining rates, (B) draining volumes, (C) pooling rates and (D) pooling volumes. We observed significantly lower draining rates (*p-value = *0.003221, N = 10) and draining volumes (*p-value = *0.001887, N = 10) in hypoxic regions as compared to normoxic regions. We observed significantly lower pooling rates (*p-value = *0.000109, N = 10) and pooling volumes (*p-value = *0.024693, N = 10) in hypoxic regions as compared to normoxic regions. Columns: Mean±SD.

Representative 3D reconstructed optical images with the hypoxic regions displayed in pink are shown in Figure 6A, along with the corresponding 3D molecular transport distributions of draining rate (Figure 6B), draining volume (Figure 6C), pooling rate (Figure 6D), and pooling volume (Figure 6E). We observed that the draining (or efflux) rates in hypoxic regions were significantly lower than those in normoxic regions (*p-value* = 0.003221, N = 10, Figure 7A), and the draining volume was significantly lower in hypoxic regions compared to normoxic regions (*p-value* = 0.001887, N = 10, Figure 7B). Draining values are negative since the MMCA leaves the voxel. The pooling (or influx) rate was significantly lower in hypoxic regions compared to normoxic regions (*p-value* = 0.000109, N = 10, Figure 7C), and the pooling volume was significantly lower in hypoxic regions as compared to normoxic regions (*p-value* = 0.024693, N = 10, Figure 7D).

**Figure 8:**
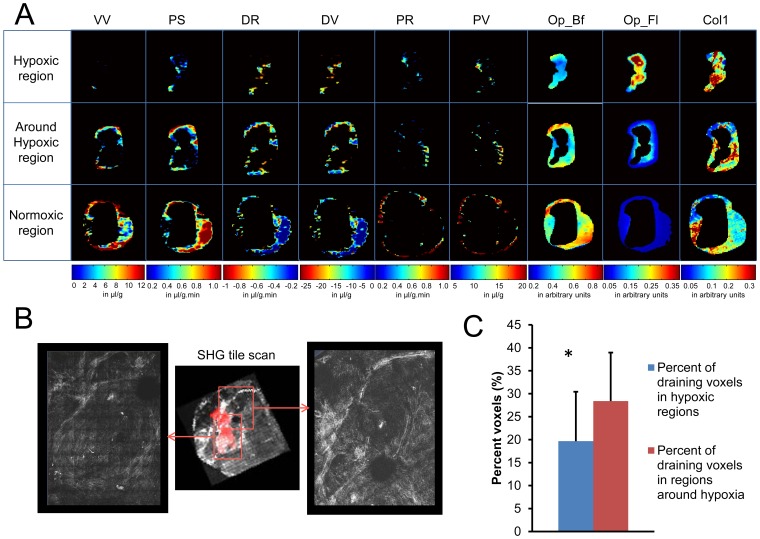
Regional Analysis. (A) Displays of the distribution of vascular parameters (VV, PS), transport parameters (DR – draining rate, DV – draining volume, PR – pooling rate, PV – pooling volume) and optical imaging parameters (OP_Bf – optical bright field, OP_Fl – optical fluorescence field, Col1– Col1 fibers) in hypoxic regions (top panel), ∼100 µm around hypoxic regions (middle panel), and in normoxic regions (bottom panel) of a representative tumor section from a breast cancer xenograft. (B) Tile scan of SHG microscopy of Col1 fibers of the same section, showing Col1 fiber patterns in the magnified inserts. (C) Quantifications of draining voxels in percent showing a significant increase (*p-value = 0.014329, N = 10) in percent draining voxels in areas surrounding hypoxic regions.

Figure 8 shows the analysis of different tumor regions. In Figure 8A, the 2D spatial distribution of VV and PS, draining rate (DR), draining volume (DV), pooling rate (PR), pooling volume (PV), optical bright field (OP_Bf), optical fluorescence field (OP_Fl) and fibers are shown for a representative tumor section in hypoxic (top panel), ∼100 µm around hypoxic (middle panel), and normoxic (bottom panel) regions. Figure 8B displays a representative SHG tile image scan with enlarged inserts showing the Col1 fiber patterns adjacent to hypoxic regions. This circular pattern around hypoxic regions was observed in most tumors. As shown in Figure 8C, MMCA movement in regions adjacent to strongly hypoxic regions increased significantly (*p-value = *0.014328) as compared to hypoxic regions.

**Figure 9:**
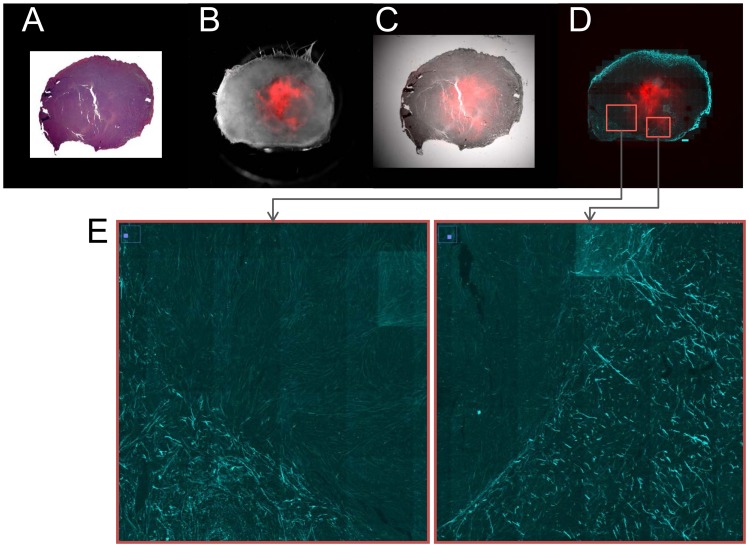
Histological analysis. (A) Hematoxylin and eosin (H&E) stained 5 µm thick section from a representative tumor, (B) corresponding overlaid bright field and fluorescence field images showing the tdTomato red fluorescence hypoxic regions, (C) overlay of H&E section with fluorescence field image, (D) overlay of corresponding SHG tile scan of Col1 fibers with fluorescence field image, and (E) magnified inserts from (D) displaying the Col1 fibers encircling the hypoxic regions.

Since chronic or acute hypoxia will ultimately result in cell death and necrosis if the tissue is not rescued by revascularization, it was important to establish that the differences in macromolecular transport between hypoxic and normoxic observed were not primarily due to severely hypoxic red fluorescing regions consisting of largely dying regions. As shown in Figures 9A–C, red fluorescing regions contained viable cells. Trends in macromolecular transport were similar irrespective of the extent of necrosis in these regions. The presence of a ring of Col1 fibers around these red fluorescing regions (Figure 9D) was again confirmed in the magnified high-resolution tiled images shown in Figure 9E.

## Discussion

Here, for the first time, we combined MRI with SHG microscopy to relate differences in collagen fibers between hypoxic and normoxic tumor regions to macromolecular fluid transport. Our purpose was to understand the role of hypoxia in modifying macromolecular fluid transport, using MRI, and collagen fiber distribution, using SHG microscopy. Higher MMCA draining voxels were observed in normoxic regions which also exhibited a dense mesh of collagen fibers. In contrast, there were few draining voxels in hypoxic regions which also exhibited fewer and structurally altered collagen fibers. These results suggest that collagen fibers may facilitate MMCA transport in tumors, for the size (∼90 kDa) and charge of the agent used here, and their absence in hypoxic regions may reduce this transport.

For these studies, we developed an imaging pipeline to extract functional and structural information from the tumor microenvironment and quantify this information. From the MRI results, we observed a heterogeneous distribution of VV, PS, draining rate and volume, and pooling rate and volume, consistent with previous observations [19]. Our data here are consistent with our previous study using the PC-3 human prostate cancer xenograft model where we observed significantly lower vascular volume in hypoxic tumor regions [18]. In that study we did not observe a significant difference in permeability between hypoxic and normoxic tumor regions. Here, consistent with earlier observations, we observed that regions with high permeability did not have high vascular volumes as is evident in Figures 4C and 8A. However, when using hypoxic and normoxic regions as classifiers, median vascular volume and permeability were significantly lower in hypoxic compared to normoxic regions. Also consistent with previous observations, we observed that the Col1 fiber distribution was heterogeneous within the tumor [9]. In severely hypoxic regions the Col1 fiber volume was significantly lower and the Col1 inter-fiber distance was significantly greater as compared to normoxic regions, as previously described [9].

Since we used FCM clustering to extract and match features from MRI and optical imaging, it was necessary to validate the registration technique for these multimodality imaging data. We therefore calculated the registration error using the Dice similarity index. The registration was only considered acceptable for cases in which the Dice similarity index was greater than 0.85. Both elastic and non-rigid registration [28], [29] were also evaluated. However, in our case, the internal features were distorted by these techniques, which caused a loss of information as was evident from the low Dice similarity index. We therefore used rigid affine transformation for the registration of the optical and MRI data, which resulted in an overlap of greater than 85% (N = 10) for the Dice similarity index. Dice similarity index values greater than 85% of overlap were considered successful for multimodality co-registration.

Higher MMCA draining rates and draining volumes were observed in normoxic regions that also exhibited a denser mesh of collagen fibers. In contrast, there were lower draining rates and draining volumes in hypoxic regions that also exhibited fewer and structurally altered Col1 fibers. Higher MMCA pooling rate and pooling volume were observed in the normoxic regions as compared to the hypoxic regions. These data suggest that hypoxic areas are like ‘silent zones’ with very little movement of MMCA as detected by this method. These data have significant implications in the transport of macromolecular therapeutic agents as they suggest that there is very little macromolecular trafficking in the ECM in hypoxic regions. This is most likely a combination of the low vascular volume and permeability resulting in low delivery as well as the sparse collagen fibers that seem to mediate transport.

In tumors such as pancreatic cancer, the dense mesh of Col1 fibers has been reported to act as a barrier to drug delivery, and Col1 depletion treatments are being developed to improve delivery [15], [30]–[32]. This fibrosis, which is typically observed in the desmoplastic stroma of pancreatic cancer, is very dense compared to what we observed in this human breast cancer xenograft model, and to what is typically observed in human breast cancers [16]. A Col1 fiber density of 25%, which we observed in normoxic regions, provides sufficient space for macromolecular transport, and in fact, our data suggest that these Col1 fibers act as guiding paths for the MMCA in the extracellular space. Since we are measuring phenomena at different spatial scales, we cannot comment on the relationship between sub-voxel variations in Col1 density and the corresponding macromolecular transport.

Immediately adjacent to the strongly hypoxic regions, a denser Col1 fiber network was observed together with increased macromolecular movement. Cancer cells have been previously observed to travel along aligned fibers [7]. The dense fiber patterns seen around the hypoxic regions may provide avenues for hypoxic cells that have several invasion genes upgregulated, to escape from this hostile environment. HIF stabilization under hypoxia transcriptionally activates several genes such as urokinase-type plasminogen-activator receptor (uPAR), matrix metalloproteinase-2 (MMP-2), autocrine motility factor (AMF), transforming growth factor-α (TGF-α), cathepsin-D (CATHD), fibronectin 1 (FN1), keratin 14 (KRT14), keratin 18 (KRT18), keratin 19 (KRT19), vimentin (VIM), c-MET tyrosine kinase that regulate invasion [2]. Col1 fibers have been shown to provide avenues for malignant cancer cells to travel along during their metastatic journey [7], [16], [33]. Cancer cells can rapidly traverse through tumors along linear Col1 fibers [33]. Some of these fibers converge with blood vessels, which can promote intravasation in the metastatic cascade [33]. Integrins such as α2β1 facilitate breast cancer cell attachment to Col1 fibers [34]. Provenzano *et al*. [7] have observed that cancer cells travel along radially aligned Col1 fiber to metastasize, and that more aggressive tumor types have characteristic tumor-associated Col1 structures (TACS). The TACS classified as TACS 3 display aligned Col1 fibers that are oriented perpendicular to pockets of tumor cells, and act as guiding paths for cancer cells on their metastatic journey [7], [35]. A study with 196 patients demonstrated that TACS 3 are predictors of survival in human breast cancer patients [35]. It was also observed that the Col1 fiber density was 45% higher in lymph node positive (LN+ve) human samples as compared to the lymph node negative (LN-ve) tumor samples, and the Col1 fiber distribution in the LN+ve patient samples was more densely packed than in the LN-ve patient samples [16]. The present study adds to an emerging picture that the stimulus for invasion and migration occurs from hypoxia, and the Col1 fibers provide avenues to escape from a hostile environment.

In summary, we have co-registered and quantified *in vivo* MRI and *ex vivo* optical imaging data to obtain structural and functional information of the tumor microenvironment in a human breast cancer xenograft model. These data highlight the importance of hypoxia and Col1 fibers in macromolecular transport. Further investigations into the role of the ECM and mechano-transduction pathways as well as cancer cell/fibroblast interactions and Col1 deposition under hypoxic conditions are merited to understand the role of the tumor microenvironment in ECM mediated drug delivery and metastasis.

## Supporting Information

Figure S1 **Establish tumor orientation.** (A) An anesthetized mouse shown with agarose gel embedding. Liquid agarose was poured into the cylindrical holder around the tumor which solidified at room temperature. The entire tumor was embedded in agarose gel and asymmetrical cuts were made in the surrounding agarose gel before acquiring the MR images. (B) M_0_ MRI maps of cross-sectional images of the tumor. The asymmetrical cut (shown by red arrows) is seen in all the M_0_ maps of the tumor.
(PPTX)
